# A novel data mining method to identify assay-specific signatures in functional genomic studies

**DOI:** 10.1186/1471-2105-7-377

**Published:** 2006-08-14

**Authors:** Derrick K Rollins, Dongmei Zhai, Alrica L Joe, Jack W Guidarelli, Abhishek Murarka, Ramon Gonzalez

**Affiliations:** 1 Department of Chemical and Biological Engineering, Iowa State University, Ames, Iowa 50011, USA; 2 Department of Statistics, Iowa State University, Ames, Iowa 50011, USA; 3 Department of Chemical and Biomolecular Engineering, Rice University, Houston, Texas 77251-1892, USA

## Abstract

**Background:**

The highly dimensional data produced by functional genomic (FG) studies makes it difficult to visualize relationships between gene products and experimental conditions (i.e., assays). Although dimensionality reduction methods such as principal component analysis (PCA) have been very useful, their application to identify assay-specific signatures has been limited by the lack of appropriate methodologies. This article proposes a new and powerful PCA-based method for the identification of assay-specific gene signatures in FG studies.

**Results:**

The proposed method (PM) is unique for several reasons. First, it is the only one, to our knowledge, that uses *gene contribution*, a product of the loading and expression level, to obtain assay signatures. The PM develops and exploits two types of assay-specific contribution plots, which are new to the application of PCA in the FG area. The first type plots the assay-specific gene contribution against the given order of the genes and reveals variations in distribution between assay-specific gene signatures as well as outliers within assay groups indicating the degree of importance of the most dominant genes. The second type plots the contribution of each gene in ascending or descending order against a constantly increasing index. This type of plots reveals assay-specific gene signatures defined by the inflection points in the curve. In addition, sharp regions within the signature define the genes that contribute the most to the signature. We proposed and used the curvature as an appropriate metric to characterize these sharp regions, thus identifying the subset of genes contributing the most to the signature. Finally, the PM uses the full dataset to determine the final gene signature, thus eliminating the chance of gene exclusion by poor screening in earlier steps. The strengths of the PM are demonstrated using a simulation study, and two studies of real DNA microarray data – a study of classification of human tissue samples and a study of *E. coli *cultures with different medium formulations.

**Conclusion:**

We have developed a PCA-based method that effectively identifies assay-specific signatures in ranked groups of genes from the full data set in a more efficient and simplistic procedure than current approaches. Although this work demonstrates the ability of the PM to identify assay-specific signatures in DNA microarray experiments, this approach could be useful in areas such as proteomics and metabolomics.

## Background

The availability of gene structure data for many organisms [1] has paved the way for the challenging task of assigning biological functions to each individual gene, and more challenging still, explaining the highly complex metabolic and regulatory networks inside the living cell, where genes, proteins, and metabolites all interrelate. The wealth of information and technologies created by the availability of these genome sequences ushered in what frequently is called the post-genomic era, along with the appearance of a new field called functional genomics (FG). FG refers to "the development and application of system-wide experimental approaches to assess gene function by making use of the information and reagents provided by structural genomics" [2]. There are at least three areas of FG for which experimental techniques are currently well developed: transcriptomics, proteomics, and metabolomics. Using a combination of these techniques with mathematical and computational tools for data analysis, the cell transcriptome, proteome, and metabolome can be identified (which refers to the inventory of all transcripts, proteins, and metabolites, respectively). Typical studies in these areas consist of surveying the levels of these species under a variety of environmental conditions and/or genetic backgrounds (referred to as assays in this article). The ultimate goal of these studies is therefore the identification of assay-specific signatures in the surveyed domain; e.g. which transcripts, proteins, or metabolites are associated with a given physiological condition such as health or disease or the response of an organism to an environmental challenge.

By far the most valuable experimental technique in FG is DNA microarrays, which have been used to study the transcriptional response of many organisms to genetic and environmental perturbations [3]. As with any other experimental tool in FG, the use of DNA microarrays to study gene expression results in large data sets, which can consist of measurements for thousands of genes. In contrast, the number of assays is typically less than hundred. Therefore, since the number of genes is much greater than the number of assays, efficient information extraction and dimensionality reduction methods are needed to obtain assay-specific gene signatures of ranked order, which is the objective of the method proposed in this work. Several statistical methodologies have been proposed to achieve this goal including principal component analysis (PCA) – a specific case of singular value decomposition (SVD), multidimensional scaling, cluster analysis, self-organizing maps, and Fisher discriminant analysis ([3] and references therein). Among them, PCA has been widely used not only in the areas of transcriptomics [4-6], but proteomics [7-9] and metabolomics [10-12] as well, and it has shown much promise in the fulfillment of our classification objective [13].

This article introduces a new and powerful PCA-based approach that differs from current PCA approaches in several critical ways. First, our proposed method (PM) determines and exploits *assay-specific gene contribution *from the complete set of PCs, that is, both eigenassays (EG) and eigengenes (EA) PCs [14]. To our knowledge this is the first application of an assay-specific gene contribution approach in this context. The score for gene *i *obtained from an EG is equivalent to contribution over all the assays for this gene. However, we are not aware of work in this context that uses a predefined subset of the score to obtain the signature as we do in this work. In contrast, for EA, gene contribution is not related to an EA score, and thus, a totally new application. Secondly, in contrast to current PCA methods, the PM does not rely on the existence of structure (i.e., gene clustering) in two dimensional score plots of dominant principal components (PCs) because they can be weak or even absent. Thirdly, this is the only PCA method, to our knowledge, that determines gene-ranked assay-specific signatures in a final step from the set of all genes, in contrast to methods that use reduced sets that have the possibility of removing critical genes at some intermediate step. This new PCA-based approach is presented using the following outline. The next section discusses concepts of PCA important to the understanding of the PM. The PM is then described in a separate section. Finally, three case studies are presented aiming at: (1) demonstrating the application and strengths of the PM along with limitations of current methods; (2) comparing the PM to an approach with similar features on an actual data set; and (3) applying the PM to study a dataset not explored before with this type of tool. Concluding remarks are given in the final section.

### A DNA microarray context for PCA

The purpose of this section is to discuss fundamental concepts of PCA in the context of microarray gene expression data that are important to the understanding of the PM. In a typical DNA microarray experiment the expression level of *m *genes is surveyed for *n *environmental conditions and/or genetic backgrounds (called assays in this article following the work of Wall *et al *[14]). We define **X **as an *m *by *n *matrix of expression data with the *n *assays expressed along the columns and the *m *genes expressed along the rows with *m *> > *n *Thus, *x*_*ij *_is the expression level of the *i*^th ^gene in the *j*^th ^assay. For convenience of discussion we let the rank of **X **= *n*. In this article, the *variables *of any matrix are expressed along the columns and the *measurements *are expressed along the rows. Thus, for **X**, the *variables *are the assays and the genes are the *measurements*. In contrast, for **X**^T^, the *variables *are genes and the *measurements *are the assays.

**Figure 1:**
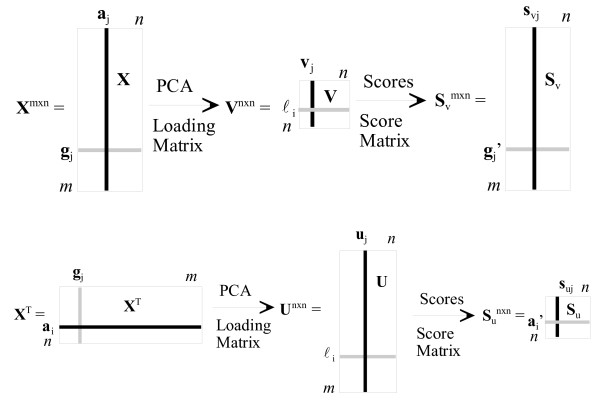
Visual representation of the data, loading, and score matrices for **X **and **X**^T^. A typical DNA microarray experiment is represented in which the expression level of *m *genes (g) is surveyed for *n *environmental conditions and/or genetic backgrounds (called assays, a).

PCA on **X **has the effect of obtaining an *n *dimensional orthogonal space such that the composite variability (i.e., change) of the genes is maximized in the first principal direction, and maximized in the second principal direction, with the variability captured in the first principal direction removed, and so on, until the last principal direction has the smallest variability. The *n *principal components (PCs) can be determined from either the covariance matrix or the correlation matrix of the *variables *of **X**. (This work uses only the correlation matrix.) The PCs are the eigenvectors of the covariance (scaled sums of squares and cross products) or the correlation (sums of squares and cross products from standardized data) matrix, and are ranked by their corresponding eigenvalues with the largest one corresponding to the first principal component (PC1). The ratio of eigenvalue *i *to the sum of all the eigenvalues is the proportion of the total variability captured by the *i*th PC. Note that the *n *PCs based on **X **have dimensions *n *by 1 (i.e., *n *elements) and following Alter *et al*. [15] they will be called eigengenes (EGs) in this article to distinguish them from the PCs based on **X**^T^, which will be called eigenassays (EAs) following Wall *et al*. [14] who departed from the "eigenarray" terminology introduced by Alter *et al*. [15]. Note that the EAs have dimensions *m *by 1. The elements (i.e., coefficients) of a PC are called *loadings*. A PC is a linear combination of the variables and the projection of the *i*th row of measurements onto a PC (i.e., the scalar product) gives its *i*th score. Let **V **(*n *by *n*) and **U **(*m *by *n*) be the loading matrices for **X **and **X**^T^, respectively. Also, let **S**_v _(*m *by *n*) and **S**_u _(*n *by *n*) be the score matrices for **X **and **X**^T^, respectively. Figure 1 illustrates these concepts and matrices visually. As shown, the EG and EA are given by **v**_j _and **u**_j_, respectively. Also note that, following Wall *et al*. [14], **g**_i _is the transcriptional response of the *i*th gene and the **a**_j_'s are the assay expression profiles, *j *= 1, . . ., *n*. Next we present the PM using the notation and description shown in Figure 1.

## Results and discussion

### Proposed method (PM)

In microarray data studies, the assay profiles are part of the experimental design. More specifically, most DNA microarray studies seek to identify assay-specific signatures (i.e., which genes determine a given phenotype as expressed by the assay). The experimenter chooses the assays because of natural relationships, a hypothesis, or both. Consequently, a considerable amount of á priori knowledge is known about the assay profiles before any analysis on the data. However, often times hidden relationships that advance knowledge are brought to light by PCA. The PM exploits the expected assay profile behavior and new knowledge to obtain assay-specific gene expression profiles or signatures with a ranking of all the genes in a profile. Our PM seeks to accomplish this objective using the following basic steps after obtaining the PCs (i.e., EGs and EAs):

1. Determine the EGs for **X **(**V**) and EAs for **X**^T ^(**U**) based on their correlation matrices.

2. Determine the pseudo score matrix for EA (**S**_u_) using **U **in Step 1 and **X**, i.e., **X**^T^**U**. **X **is not standarized.

3. Plot the EG loadings (the row coefficients of **V**) and pseudo EA scores (the row coefficients of **X**^T^**U**) against the assay index starting from the most dominant to the lesser dominant PCs until the assay signature of interest is revealed as a group of large outliers.

4. Determine the contribution for each gene using the selected PC in Step 1 for the assay signature group.

5. Rank the genes by contribution and plot the contributions in ranked order against an index that increases by one unit for each gene. Gene identification with rank should be maintained.

6. Obtain the assay-specific gene signatures from the ranked assay profiles using the plot in Step 3 to determine cut-offs or limits on the size of profile groups.

Most PC software packages give an option for selecting between the covariance or correlation matrix of the variables. For standardization of scale, we recommend using the correlation option in Step 1. However, for obtaining the score matrices in Step 2, our procedure is to standardize **X **for obtaining EG scores but not for EA scores since we do not standardized **X **when determining EA gene contribution. Even though the EA are determined from the correlation matrix, we do not standardize **X **because under EA standardization **X **is row centered and since the contribution of each gene *i *is proportional to the sum across row *i*, these contribution values tend to disappear under standardization. The EG standardization of **X **is given below:

Z=[z1z2⋯zn]=[x11−x¯1s1x12−x¯2s2⋯x1n−x¯nsnx21−x¯1s1x22−x¯2s2⋯x2n−x¯nsn⋮⋮⋮xm1−x¯1s1xm2−x¯2s2⋯xmn−x¯nsn]     (1)
 MathType@MTEF@5@5@+=feaafiart1ev1aaatCvAUfKttLearuWrP9MDH5MBPbIqV92AaeXatLxBI9gBaebbnrfifHhDYfgasaacH8akY=wiFfYdH8Gipec8Eeeu0xXdbba9frFj0=OqFfea0dXdd9vqai=hGuQ8kuc9pgc9s8qqaq=dirpe0xb9q8qiLsFr0=vr0=vr0dc8meaabaqaciaacaGaaeqabaqabeGadaaakeaaieqacqWFAbGwcqGH9aqpdaWadiqaaiab=Pha6naaBaaaleaacqaIXaqmaeqaaOGae8hiaaIae8NEaO3aaSbaaSqaaiabikdaYaqabaGccqWIVlctcqWF6bGEdaWgaaWcbaGaeeOBa4gabeaaaOGaay5waiaaw2faaiabg2da9maadmGabaqbaeqabqabaaaaaeaadaWcaaqaaiabbIha4naaBaaaleaacqaIXaqmcqaIXaqmaeqaaOGaeyOeI0IafeiEaGNbaebadaWgaaWcbaGaeGymaedabeaaaOqaaiabbohaZnaaBaaaleaacqaIXaqmaeqaaaaaaOqaamaalaaabaGaeeiEaG3aaSbaaSqaaiabigdaXiabikdaYaqabaGccqGHsislcuqG4baEgaqeamaaBaaaleaacqaIYaGmaeqaaaGcbaGaee4Cam3aaSbaaSqaaiabikdaYaqabaaaaaGcbaGaeS47IWeabaWaaSaaaeaacqqG4baEdaWgaaWcbaGaeGymaeJaeeOBa4gabeaakiabgkHiTiqbbIha4zaaraWaaSbaaSqaaiabb6gaUbqabaaakeaacqqGZbWCdaWgaaWcbaGaeeOBa4gabeaaaaaakeaadaWcaaqaaiabbIha4naaBaaaleaacqaIYaGmcqaIXaqmaeqaaOGaeyOeI0IafeiEaGNbaebadaWgaaWcbaGaeGymaedabeaaaOqaaiabbohaZnaaBaaaleaacqaIXaqmaeqaaaaaaOqaamaalaaabaGaeeiEaG3aaSbaaSqaaiabikdaYiabikdaYaqabaGccqGHsislcuqG4baEgaqeamaaBaaaleaacqaIYaGmaeqaaaGcbaGaee4Cam3aaSbaaSqaaiabikdaYaqabaaaaaGcbaGaeS47IWeabaWaaSaaaeaacqqG4baEdaWgaaWcbaGaeGOmaiJaeeOBa4gabeaakiabgkHiTiqbbIha4zaaraWaaSbaaSqaaiabb6gaUbqabaaakeaacqqGZbWCdaWgaaWcbaGaeeOBa4gabeaaaaaakeaacqWIUlstaeaacqWIUlstaeaaaeaacqWIUlstaeaadaWcaaqaaiabbIha4naaBaaaleaacqqGTbqBcqaIXaqmaeqaaOGaeyOeI0IafeiEaGNbaebadaWgaaWcbaGaeGymaedabeaaaOqaaiabbohaZnaaBaaaleaacqaIXaqmaeqaaaaaaOqaamaalaaabaGaeeiEaG3aaSbaaSqaaiabb2gaTjabikdaYaqabaGccqGHsislcuqG4baEgaqeamaaBaaaleaacqaIYaGmaeqaaaGcbaGaee4Cam3aaSbaaSqaaiabikdaYaqabaaaaaGcbaGaeS47IWeabaWaaSaaaeaacqqG4baEdaWgaaWcbaGaeeyBa0MaeeOBa4gabeaakiabgkHiTiqbbIha4zaaraWaaSbaaSqaaiabb6gaUbqabaaakeaacqqGZbWCdaWgaaWcbaGaeeOBa4gabeaaaaaaaaGccaGLBbGaayzxaaGaaCzcaiaaxMaadaqadiqaaiabigdaXaGaayjkaiaawMcaaaaa@AC33@

Note that the scores matrix for EG (**S**_v_) is determined by **S**_v _= **ZV**.

The objective of Step 3 is to determine and select the PC with the strongest character of the assay group of interest using loading plots for the EGs and score plots for the EAs. These plots are compared against the á priori knowledge of the assays. In addition, these plots can also bring to light relationships among assays not known or hypothesized á priori. The strength of a specific profile is determined by its PC association, the amount of variation explained by the associated PC, and its separation from the other assay profiles. The PC associated with the plot with the strongest profile is selected to determine the signature. This can be either an EG or an EA. Our experience have found that a number of EA and EG PCs provide similar information but that either one can provide unique information. This is why we give the recommendation to examine both types of eigenvectors.

Steps 4–6 involve the development, analysis, and use of gene contribution plots. To our knowledge this is the first use of contribution plots for determining signatures. This approach has the advantage over a loading approach because the loadings only give the relative weight for a specific PC but the contribution gives the product of the relative weight and the expression level for a particular gene. In addition, our approach has the advantage of determining this contribution for only the members in the assay group of interest whereas the loading gives an effect across all the assays. The contribution CkiG
 MathType@MTEF@5@5@+=feaafiart1ev1aaatCvAUfKttLearuWrP9MDH5MBPbIqV92AaeXatLxBI9gBaebbnrfifHhDYfgasaacH8akY=wiFfYdH8Gipec8Eeeu0xXdbba9frFj0=OqFfea0dXdd9vqai=hGuQ8kuc9pgc9s8qqaq=dirpe0xb9q8qiLsFr0=vr0=vr0dc8meaabaqaciaacaGaaeqabaqabeGadaaakeaadaqfWaqabSqaaiabbMgaPbqaaiabbEeahbqdbaWaaSraa4qaaiabbUgaRbqabaqdcqqGdbWqaaaaaa@3226@ for a specified assay group, *GP*, for gene *i*, using EG **v**_k _is

CkiGP=∑Over
          j' s in GPZijVjk.     (2)
 MathType@MTEF@5@5@+=feaafiart1ev1aaatCvAUfKttLearuWrP9MDH5MBPbIqV92AaeXatLxBI9gBaebbnrfifHhDYfgasaacH8akY=wiFfYdH8Gipec8Eeeu0xXdbba9frFj0=OqFfea0dXdd9vqai=hGuQ8kuc9pgc9s8qqaq=dirpe0xb9q8qiLsFr0=vr0=vr0dc8meaabaqaciaacaGaaeqabaqabeGadaaakeaadaqfWaqabSqaaiabbMgaPbqaaiabbEeahjabbcfaqbqdbaWaaSraa4qaaiabbUgaRbqabaqdcqqGdbWqaaGccqGH9aqpdaaeqbqaaiabbQfaAnaaBaaaleaacqqGPbqAcqqGQbGAaeqaaOGaeeOvay1aaSbaaSqaaiabbQgaQjabbUgaRbqabaaabaqbaeaabiqaaaqaaiabb+eapjabbAha2jabbwgaLjabbkhaYbqaaiqbbQgaQzaafaGaee4CamNaeeiiaaIaeiyAaKMaeiOBa4MaeeiiaaIaee4raCKaeeiuaafaaaqab0GaeyyeIuoakiabc6caUiaaxMaacaWLjaWaaeWaceaacqaIYaGmaiaawIcacaGLPaaaaaa@521C@

Similarly, using EA **u**_k_,  is

CkiGP=uik∑Over
																 j's in GPXij.     (3)
 MathType@MTEF@5@5@+=feaafiart1ev1aaatCvAUfKttLearuWrP9MDH5MBPbIqV92AaeXatLxBI9gBaebbnrfifHhDYfgasaacH8akY=wiFfYdH8Gipec8Eeeu0xXdbba9frFj0=OqFfea0dXdd9vqai=hGuQ8kuc9pgc9s8qqaq=dirpe0xb9q8qiLsFr0=vr0=vr0dc8meaabaqaciaacaGaaeqabaqabeGadaaakeaadaqfWaqabSqaaiabbMgaPbqaaiabbEeahjabbcfaqbqdbaWaaSraa4qaaiabbUgaRbqabaqdcqqGdbWqaaGccqGH9aqpcqqG1bqDdaWgaaWcbaGaeeyAaKMaee4AaSgabeaakmaaqafabaGaeeiwaG1aaSbaaSqaaiabbMgaPjabbQgaQbqabaaabaqbaeaabiqaaaqaaiabb+eapjabbAha2jabbwgaLjabbkhaYbqaaiqbbQgaQzaafaGaee4CamNaeeiiaaIaeiyAaKMaeiOBa4MaeeiiaaIaee4raCKaeeiuaafaaaqab0GaeyyeIuoakiabc6caUiaaxMaacaWLjaWaaeWaceaacqaIZaWmaiaawIcacaGLPaaaaaa@5256@

Note that from Eq. (2), if *GP *consists of all the assays,  is the *i*^th ^score for the *j*^th ^EG. In contrast, note from Eq. (3),  is not related to the scores of EA.

If the absolute value of  is high, then we are assuming that it is critical to the assay-specific signature and the greater , the more critical the gene. Thus, by ranking the genes from the highest  to the lowest , we are able to obtain a ranked list of genes for each assay-specific signature. The cut-off point is guided by the level of change across the gene index revealed in the ranked contribution plots. Nonetheless, since the genes are ranked, no matter where one makes the cut-off, the list will be the strongest one for the selected number of genes. We now apply the PM to three cases; a simulation study of artificial data and two cases of real data and demonstrate its ability to obtain assay-specific gene signatures of ranked order.

### The case studies

In this section we present three case studies to evaluate our PM. The first study uses simulated data based on the study of Wall *et al*. [14]. This study will validate the PM by demonstrating its ability to obtain the correct assay-specific gene signatures. The second study involves applying the PM to a real DNA microarray data set (the case studied by Misra *et al*. [13]). This study consisted of 6,972 genes and 40 normal human tissue samples, which they used to develop and apply a novel method for obtaining tissue-specific gene expression signatures. In this case study we discuss the limitations of their method as compared to the strengths of the PM and compare results. The last study uses real expression data of 4,290 identified genes and twelve assays, representing a combination of two recombinant *E. coli *strains and different cultivation conditions including the alternative use of two sugars and the exposure of the cells to different ethanol concentrations (the case studied by Gonzalez *et al*. [16]). We analyze this data set using our PM and present gene expression profiles for the most relevant assay-specific signatures: i.e., the response of the cells to a 2% ethanol challenge.

#### The simulation data study

**Figure 2:**
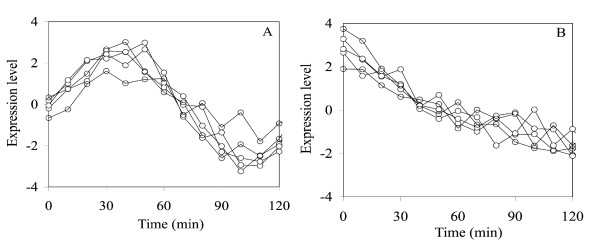
Five gene transcriptional responses from the noisy sign data (A) and the noisy exponential data (B) to verify agreement with the simulated data produced by Wall *et al*. [14].

**Figure 3:**
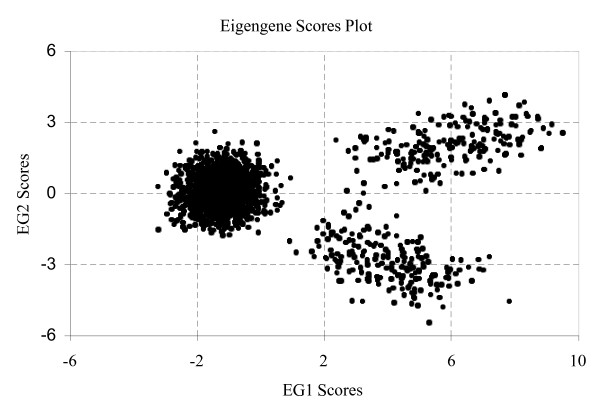
A score scatter plot of EG2 vs. EG1 for the simulated data. The genes separate into three cluster groups but this plot does not provide knowledge of gene or assay characteristics.

This study generates artificial gene expression data mimicking the simulation study in Wall *et al*. [14] to introduce and illustrate the PM. This data have three kinds of transcriptional responses: 1. noisy (genes 1 to 1,600); 2. noisy sinusoidal behavior (genes 1601 to 1,800); and 3. noisy exponential pattern (1,801 to 2,000). The added noise was distributed normally with mean 0 and standard deviation 0.5. The sine pattern was



where *a *is distributed uniformly on the interval (1.5, 3). The exponential pattern was

be−t100     (5)
 MathType@MTEF@5@5@+=feaafiart1ev1aaatCvAUfKttLearuWrP9MDH5MBPbIqV92AaeXatLxBI9gBaebbnrfifHhDYfgasaacH8akY=wiFfYdH8Gipec8Eeeu0xXdbba9frFj0=OqFfea0dXdd9vqai=hGuQ8kuc9pgc9s8qqaq=dirpe0xb9q8qiLsFr0=vr0=vr0dc8meaabaqaciaacaGaaeqabaqabeGadaaakeaaieqacqWFIbGycqWFLbqzdaahaaWcbeqaaiabgkHiTmaalaaabaGae8hDaqhabaGaeGymaeJaeGimaaJaeGimaadaaaaakiaaxMaacaWLjaWaaeWaceaacqaI1aqnaiaawIcacaGLPaaaaaa@3882@

where *b *is distributed uniformly on the interval (4, 8) and *t *is the time (in minutes). The number of assays is fourteen with assay *i *corresponding to sampling at time 10*i *min, *i *= 0, 1, . . ., 13. To verify that our data were in agreement with the Wall *et al*. [14] data, we plotted the graphs in Figure 2. These plots are in agreement with their Figure 5.3. As we discussed in previous section, a two-dimensional score scatter plot based on EG1 and EG2 is common in PCA analysis. Although Figure 3 separates three clusters of genes in agreement with the nature of this data set, it does not provide knowledge of the gene or assay characteristics for the clusters and is therefore not used by the PM. In this study we are assuming that the experimenter has designed the sinusoidal and exponential patterns into the assays. Thus, the experimenter has a complete knowledge of this behavior. For space considerations we will not show these plots but they would look like the ones in Figure 2, except that there would be one point plotted for each assay number. The PM will be able to effectively accomplish its objective if it can capture and reveal these patterns in a few dominant PCs, preferably PC1 and PC2.

**Figure 4:**
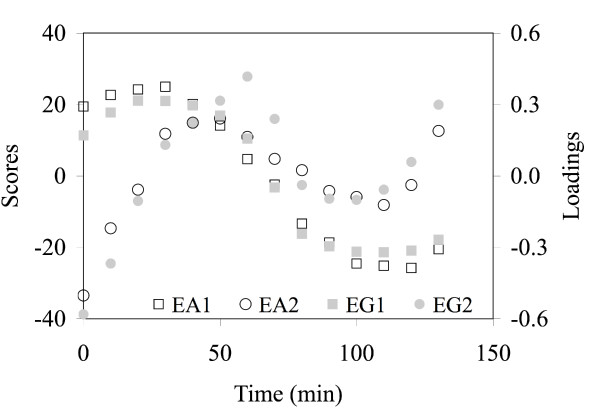
The EG loading and EA score plots versus assay (time) based on the first two PCs for the simulation data. The sinusoidal behavior is clearly shown and the exponential behavior appears to be also contained in the plots based on PC1 since the period is larger than the one shown in Figure 2.

In applying the PM, we first determine the PC matrices **U **and **V **as required by Step 1. We then determine the pseudo scores for the EAs per Step 2 and produce Figure 4 under Step 3. This figure plots the loading for EG and the pseudo scores for EA against the assay time index. Sinusoidal patterns are clearly revealed by all the curves although the one for EG2 appears to be distorted. The EA2 plot appears to match the sinusoidal behavior in Figure 2 the best. The EG1 loading plot and the EA1 score plot appear to have the right sinusoidal shape but with a larger period. Closer examination of these plots, especially the EA1 score plot, indicates evidence of a composite behavior of both patterns. EG1 and EG2 explained about 51% and 13% of the total variation, respectively, and EA1 and EA2 explained about 21% and 9%, respectively. In our research, we have found much lower amounts for EAs to be common. However, one should not use this as a criterion for choosing an EG analysis over an EA analysis. As discussed previously, the PC selection criteria that we recommend are the separation and grouping of assay groups as well as the order of the PC.

**Figure 5:**
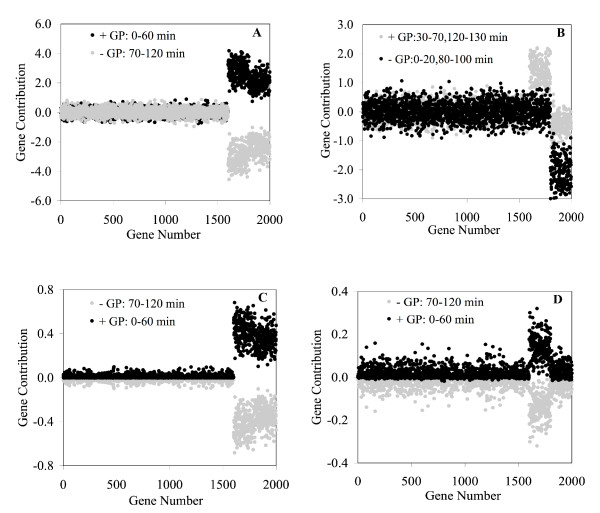
The contribution plots for EG1 (A), EG2 (B), EA1 (C), and EA2 (D). "GP" = group.

In applying Step 4, at least two types of assay profiles or groups need to be defined to identify the sinusoidal and exponential genes. For this particular case, we chose to do this by defining these groups based on positive and negative loadings and scores in Figure 4. The results of this grouping are given by the score contribution plots in Figure 5. In these plots, the contribution of gene *i*, for a given PC, is the sum of the scores for all the genes for the particular grouping (either positive or negative). EA1 plot is very effective separating both induced gene types (1,601–2,000) from the noisy genes (Figure 5C), while the EA2 plot confirms that this PC represents only the sinusoidal genes (Figure 5D), although the identification is not so distinct for a significant number of genes. Although EG1 appears to represent both gene types and does an excellent job of identifying as one group all 400 genes (Figure 5A), the EG2 score contribution plot appears to represent the sinusoidal genes for the positive (+) assay group and the exponential genes for the negative (-) assay group (Figure 5B). In summary, the EA analysis is a better choice for this data set than an EG analysis since it better identifies both types of genes using EA1 and sinusoidal genes using EA2.

**Figure 6:**
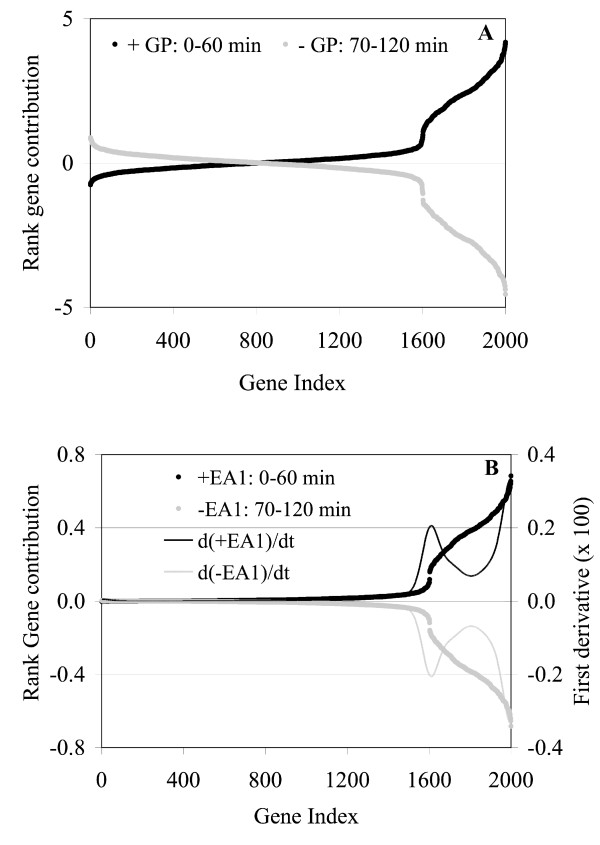
Rank gene contribution plots for EG1 (A) and EA1 (2). "GP" = group. A sharp change occurs at index 1,600 with a gradual change approaching 2,000 in agreement with reality. EA is the better plot as seen by its low gene ranking behavior in agreement with reality.

After separating the genes by their PC assays groups, Step 5 is applied. The objective of this step is to develop a ranked-order list for all the groups identified in Step 4. To do this, we again use the contributions obtained in Step 4 and appearing in Figure 5 but now we plot them in ascending or descending order against an index that increases by one unit for each gene. The ranked gene plots for both EG and EA results are given in Figure 6. Both plots look very similar except for low index numbers. For the EG plot, the lines for the two groups cross and separate, thus creating a false gene signature for the lower ranking genes (i.e., this is not true since they are all noisy genes). The EA plot however does not create such a false signature since both lines are close to zero as the rank decreases and is thus the better choice here also. As the rank increases, the shape of the curves permit the identification of two groups of genes clearly separated by two inflection points around gene numbers 1,600 and 1,800. These inflection points can be obtained by identifying the optimum (minimum or maximum) of the first derivative. We show this calculation for the EAs plot in Figure 6 and, as expected, two inflection points are observed at gene numbers 1,600 and 1,800. This feature of the gene contribution plots can be used to precisely define complete sets of genes that represent an assay signature. It is noteworthy to mention that the only sharp changes in this curve take place in the transition from the noisy to induced genes thus clearly indicating the start of the signature (i.e., induced genes). No sharp breaks occurred within the region of induced genes, indicating the truth that no gene in this expression signature is dominant.

#### Human tissue expression data case study

Our second study compares the results and methodology of the PM with another one (Misra *et al*. [13], referred to as the "Misra *et al*." method in what follows) of similar ability (i.e., a PCA-based method) in a study of real microarray data. Their approach represents the most refined use of PCA for the analysis of FG data that we found in the literature. This data set consists of assays from several human tissues from the brain, kidney, lung, esophagus, skeletal muscle, breast, stomach, colon, blood, spleen, prostate, testes, vulva, proliferative endometrium, myometrium, placenta, cervix and ovary [see Additional file 1]. We will first briefly describe their approach and compare and contrast it to the PM. Then we apply the PM and determine tissue-specific gene expression signatures for brain, liver, and muscle assays. We close this section by comparing the signature results of both methods.

Although it is not clear from their loading and score plots, we determined that the PCA method of Misra *et al*. is based on eigengenes (EGs). Their method is basically a gene screening method where genes are removed at various reduction steps while seeking to maintain the structure of the loading plot. Their first reduction step is a course screening and reduces the total set of genes to a workable subset (from about 7,000 genes to 425) using a filtering method that eliminates genes with loadings below a threshold. One limitation is that this threshold is not based on statistical significance but is somewhat subjective, although quantitative. This set of genes is then reduced to unrefined signatures without identities at this point using a histogram and visually determining the classes (i.e., the gene groups). These unrefined gene groups are refined (i.e., further reduced) using cluster analysis and visually eliminating genes outside subjectively chosen cluster groups. The final step uses score plots to "reveal" the "nature of each gene group" and to rank the genes within each group.

The Misra *et al*. method is similar to our PM in that the objective is to identify signature groups and prioritize the genes in these groups. However, there are some very critical differences. First, they only make use of EGs in contrast to the PM that compare EGs to EAs and selects the one that best represents the assay group of interest. Another critical difference is that their method relies on structure in two dimensional loading plots but the PM does not since, as we determined by simulation (previous section) and will show in the ethanol study (next section), this type of plot will not always separate genes into identifiable groups (i.e., have useful structure). Furthermore, for the Misra *et al*. method, gene contribution is based on the loading of a gene whereas in the PM gene contribution is the sum of the product of the loading and the expression level for only the assays in the signature group (see Eqs. 2–3). Another critical difference is that when the Misra *et al*. method ranks the genes within a gene group only the "refined" genes are candidates; and thus, not giving a chance to those not in this group that could have been incorrectly eliminated. In contrast, when the genes are ranked by the PM all the genes are considered. Finally, the PM seems to be much simpler to apply as we now demonstrate on this study.

**Figure 7:**
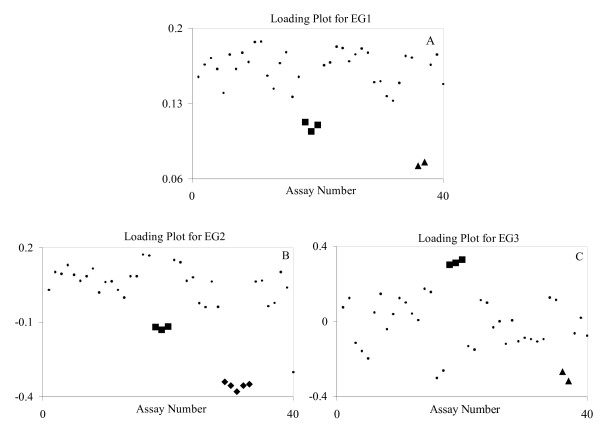
Loading plots for EG1 (A), EG2 (B), and EG3 (C) for the human tissue study. The squares are the muscles group, the diamonds are the brain group, and the triangles are the liver group. Although these groups were clearly defined, EA2 and EA3, as shown in Figure 8, had better separation and were used to develop these group signatures.

**Figure 8:**
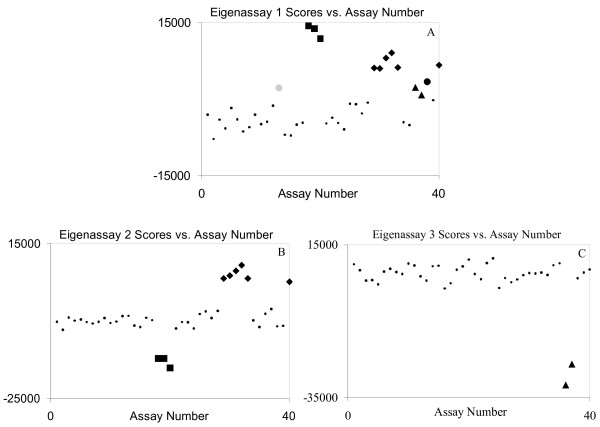
Score plots for EA1 (A), EA2 (B), and EA3 (C) for the human tissue data. The large gray dot is Placenta 2, the squares are the muscles group, the diamonds are the brain group, the large black dot is Breast 9, and the triangles are the liver group. EA2 and EA3 were chosen to develop the muscle, brain, and liver signatures.

Figure 7 gives the loading plots for EA1–EA3 and Figure 8 gives the score plots for EA1 to EA3. EG1–EG3 explained about 60%, 7%, and 6% of the total variation, respectively, and EA1–EA3 explained about 12%, 8% and 7%, respectively. EG1 separates the muscle and liver assays well, the only drawback is that they are on the same side of the cluster of points. This drawback is also true of EG2 in the separation of the muscle and brain assays. However, this is not true for EG3 in the separation of the muscle and liver assays but the drawback of this plot is that the separations are not very prevalent. We did not choose any of these PCs for signature definition because we found some EAs with better properties.

Figure 8 contains the score plots for the first three EAs. For the EA1 plot, from the positive values, the first PC appears to reflect a combination of Placenta 2 (gray dot), the three muscles (squares), the six brain (diamonds), Breast 9 (the large black dot), and the two liver (the triangles) tissues. All these groups are on the same side of the cluster and, thus we did not use this PC for assay development. In contrast, EA2 separates nicely into muscle and brain assays on opposite sides of the cluster. Similarly, EA3 separates nicely into only a liver assay group. Hence, we selected EA2 to develop the muscle and brain signatures and EA3 to develop the liver signatures.

**Figure 9:**
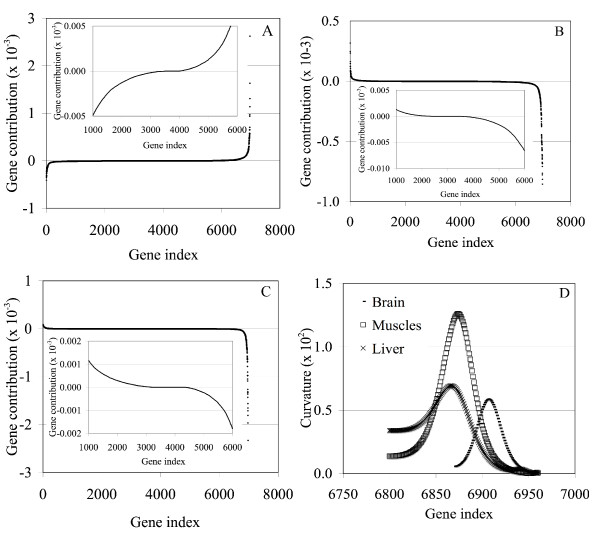
The ranked gene contribution plots for the brain (A), muscles (B), and liver (C) gene groups. The insets show the inflection point of each curve, which defines the whole set of genes composing the signature. The changes are very sharp as the highest-ranking genes are approached, which allows the definition of a subset of genes contributing the most to the signature. The cut-off values were determined by identifying the point of maximum curvature in these regions. Panel (D) shows the change in curvature for the steepest region of each curve represented in panels (A)-(C).

The contribution plots ranking the genes within the signatures using EA2 and EA3 are shown in Figure 9A–C. As one can see, the EAs produced well defined signatures with sharp, steep rises that significantly distinguish contributions for the most dominant genes. As in the simulation case study, an inflection point can be identified which marks the start of a tissue-specific signature (see insets in Figure 9A–C). Using this approach, 3,014, 3,492, and 2,647 genes are identified as the complete brain, muscles, and liver signatures, respectively. This list is clearly too comprehensive and of limited practical use due to its length. However, because of the sharp changes over a small number of genes as the highest ranking genes are approached, a subset of genes contributing the most to each tissue-specific signature can be clearly observed.

We identified the cut-off point that marks the limit of this subset of genes by using the curvature (κ) as a metric to assess the steepness of the curve. For a function *y *= *f *(*x*), the curvature is defined as

κ=f(x)″[1+(f(x)′)2]32     (6)
 MathType@MTEF@5@5@+=feaafiart1ev1aaatCvAUfKttLearuWrP9MDH5MBPbIqV92AaeXatLxBI9gBaebbnrfifHhDYfgasaacH8akY=wiFfYdH8Gipec8Eeeu0xXdbba9frFj0=OqFfea0dXdd9vqai=hGuQ8kuc9pgc9s8qqaq=dirpe0xb9q8qiLsFr0=vr0=vr0dc8meaabaqaciaacaGaaeqabaqabeGadaaakeaacqaH6oWAcqGH9aqpdaWcaaqaaiabdAgaMjabcIcaOiabdIha4jqbcMcaPyaagaaabaWaamWaceaacqaIXaqmcqGHRaWkdaqadiqaaiabdAgaMjabcIcaOiabdIha4jqbcMcaPyaafaaacaGLOaGaayzkaaWaaWbaaSqabeaacqaIYaGmaaaakiaawUfacaGLDbaadaahaaWcbeqaamaalmaameaacqaIZaWmaeaacqaIYaGmaaaaaaaakiaaxMaacaWLjaWaaeWaceaacqaI2aGnaiaawIcacaGLPaaaaaa@450C@

When the curve is changing sharply κ is large and when the curve changes slowly κ is small. Thus, by calculating the gene index that produces a maximum value of κ, a clear cut-off in the signature can be identified. We calculated κ for the sharp regions in the ranked gene contribution plots, and in all cases there is a clear maximum defining the cut-off points (see Figure 9D). Using this approach the size of the subset of genes defining the brain, liver, and muscles signatures was 67, 107, and 99, respectively. Individual genes in the brain-specific signature are shown in Table 1, and Tables 2 and 3 show the results for the liver and muscle signatures [for complete tables, including gene function, see Additional file 2].

**Table 1:** Brain-specific signature as identified by the proposed method.

**Rank**	**Gene ID**	**Rank**	**Gene ID**	**Rank**	**Gene ID**	**Rank**	**Gene ID**
1	M63379*	18	**D21267 (8)**	35	U49869*	52	K03515*
2	**S72043 (1)**	19	D87463*	36	**M25667 (18)**	53	**D49958 (27)**
3	**M13577 (3)**	20	**D78577 (19)**	37	**L20814 (20)**	54	L18983*
4	D86974	21	**L07807 (9)**	38	D50310	55	D87460*
5	M27891*	22	X95404*	39	J05243	56	J04988
6	**S40719 (3)**	23	**L10373 (11)**	40	**HG3437-HT3628 (10)**	57	U51336*
7	**J04615 (7)**	24	X15341*	41	L11373*	58	HG4322-HT4592
8	X05196*	25	**M16364 (12)**	42	J04173	59	X51956*
9	M19311	26	M17733	43	**M11749 (24)**	60	U47634
10	**U44839 (14)**	27	**D63851 (15)**	44	**X86809 (30)**	61	M86400*
11	**U48437 (6)**	28	U04241	45	**X04741 (22)**	62	D49400
12	**X99076 (5)**	29	**Y09836 (16)**	46	**M65066 (28)**	63	X15183
13	M21142*	30	**L37033 (23)**	47	**D87465 (29)**	64	HG1862-HT1897
14	Z70759	31	M74491*	48	**S82024 (26)**	65	U60644*
15	**HG1877-HT1917 (4)**	32	S77356	49	D55654	66	S78296*
16	J03077*	33	**J04046 (21)**	50	L47738*	67	D13146*
17	**M98539 (13)**	34	**M37457 (17)**	51	**D82343 (25)**		

Genes previously identified as part of this signature by Misra *et al*. [13] are presented in bold and their ranks are shown in parenthesis. Genes marked with an asterisk are deemed to be good markers of the specific tissue because previous studies haven shown them to be either functionally associated with the health/disease state of the specific tissue or expressed at high levels on it [17].

**Table 2:** Liver-specific signature as identified by the proposed method.

**Rank**	**Gene ID**	**Rank**	**Gene ID**	**Rank**	**Gene ID**	**Rank**	**Gene ID**
1	X01038*	28	**J02843 (2)**	55	**X51441 (11)**	82	HG2841-HT2968
2	K01396*	29	M65292	56	**M21642 (15)**	83	M62486*
3	M69197	30	**M13149 (6)**	57	X76717	84	S82297
4	K02765*	31	**X03168 (8)**	58	J05428	85	X56411*
5	X02544*	32	**M10050 (7)**	59	M10612*	86	U46499*
6	M15517	33	V00594	60	U08021*	87	U77594
7	J00129	34	**HG1827-HT1856 (12)**	61	X16260*	88	X02176
8	M20902*	35	**D14446 (9)**	62	J03910	89	M75106
9	X04898	36	**M16961 (10)**	63	**M19828 (16)**	90	M33317*
10	K03431	37	M63379	64	X57351	91	L05144*
11	V00594	38	D13900*	65	**X14690 (18)**	92	X56692*
12	X01388*	39	X65727*	66	M61855*	93	M29874
13	U22961	40	M12963*	67	M14058	94	L09708
14	M12529*	41	M34276*	68	M22976*	95	J02943*
15	X00129*	42	M11437	69	M10942	96	K02402
16	L15702*	43	M13690*	70	M25079	97	Y09616
17	M15656*	44	X64177	71	U22029	98	L09229
18	S95936*	45	U21931*	72	**M20786 (20)**	99	L48516
19	M59815	46	X83618*	73	D16294	100	D78011*
20	**X53595 (3)**	47	D00408*	74	L29008	101	U51010
21	M10014	48	D87292	75	**M21642 (19)**	102	**S48983 (24)**
22	**HG2841-HT2969 (4)**	49	X53414*	76	M17262	103	X63359
23	M11147	50	**M58600 (14)**	77	**U08006 (22)**	104	L47726
24	**HG2841-HT2970 (5)**	51	X68733*	78	**M11321 (21)**	105	L07765*
25	**M36803 (1)**	52	X02761	79	**M11567 (17)**	106	K03192
26	D38535	53	**L00190 (13)**	80	X13930	107	K02766
27	J04080	54	D31628*	81	X05409*		

Genes presented in bold or marked with an asterisk and numbers in parenthesis are as described for Table 1.

**Table 3:** Muscle-specific signature as identified by the proposed method.

Rank	Gene ID	Rank	Gene ID	Rank	Gene ID	Rank	Gene ID
1	M21812*	26	HG2442-HT2538	51	M83088	76	X01677
2	**X00371 (1)**	27	M83186*	52	HG4749-HT5197	77	M63603*
3	**M33772 (2)**	28	U14973	53	X63527	78	M26880
4	X16064	29	X04201*	54	U06155	79	X15940
5	M37984*	30	**M20642 (16)**	55	HG3549-HT3751	80	D14530
6	**Z20656 (3)**	31	U60115	56	S45630	81	M60092*
7	**M21494 (4)**	32	**X16504 (15)**	57	J05073*	82	U24183*
8	**U96094 (5)**	33	Z23090	58	L19527	83	U49837*
9	M21984*	34	**U35637 (17)**	59	X06617	84	X69433
10	M17886	35	HG2873-HT3017	60	M31520	85	D23660
11	**M19309 (11)**	36	M32598*	61	M64716	86	X60036*
12	X66141*	37	X17206	62	U65581*	87	Z49878
13	**M20543 (14)**	38	**M29458 (18)**	63	J03827*	88	U14970
14	M17885	39	HG1800-HT1823	64	Z12962	89	X69654
15	**M83308 (7)**	40	X03342	65	D14710	90	M63391*
16	**S73840 (13)**	41	X80822	66	X62691	91	X16560
17	**J04760 (6)**	42	Z49148	67	X95325*	92	M18000
18	**X90568 (12)**	43	**M86407 (19)**	68	X56932	93	X51466
19	M24122*	44	X02152*	69	U57341	94	L32977
20	U96781*	45	S73591	70	HG4011-HT4804	95	D21235
21	**L21715 (9)**	46	M60854	71	X69150	96	L16842
22	**X06825 (8)**	47	D79205	72	HG3364-HT3541	97	U14968
23	**M21665 (10)**	48	X12447*	73	U29175	98	M22632
24	X73113*	49	M55409	74	L26247	99	U12465
25	X66276*	50	HG821-HT821	75	M24069*		

Genes presented in bold or marked with an asterisk and numbers in parenthesis are as described for Table 1.

The PM clearly identifies the genes reported by Misra *et al*. [13] as part of each tissue-specific signature (genes reported in their study are shown in bold in these tables with their original ranking in parenthesis). We also investigated whether the genes newly identified using the PM are indeed representative of each tissue by reviewing current information available at "Entrez Gene" (), a database from the National Center for Biotechnology Information [17] and its multiple links to the latest reports available in the literature. Genes marked with an asterisk in Tables 1, 2, 3 are deemed to be good markers of the specific tissue because previous studies have shown them to be either functionally associated with the health/disease state and/or expressed at high levels in the tissue. Clearly, a very large proportion of the newly identified genes are in fact good markers of the individual tissues. In addition, the identification of new genes constitute the basis to formulate hypothesis regarding their involvement in the functioning of the specific tissue, thus evidencing the potential use of the PM to identify new functions. For example, a very large proportion of newly identified genes in the muscle signature (see Table 3) encode ribosomal proteins, which would indicate their differential expression in this tissue. This is in fact in agreement with existing evidence that supports the tissue-specific expression of ribosomal proteins [18]. Using additional PCs (actually only EGs), we were able to develop signatures for a number of assay tissue groups. For space considerations, we do not present them in this article but for the interested reader they can be found at the website:  .

#### The ethanol response in E. coli case study

After demonstrating the superior performance of the PM in both a simulation study and a typical analysis of DNA microarray data using existing PCA methodologies (two previous sections), we now present the application of the PM to investigate a data set that had not been analyzed using any PCA-based method; i.e., the study of ethanol tolerance in ethanologenic *E. coli *strains KO11 and LY01 by Gonzalez *et al*. [16] [see Additional file 3]. The aim of their study was to identify the basis for ethanol tolerance in ethanol-resistant strain LY01 by comparison to its parent strain KO11 using genome-wide transcriptional profiling. This data set consists of 12 assays, which are a combination of two genetic backgrounds (strains KO11 and LY01) and different variations of cultivation conditions such as presence/absence of ethanol (as 1% in the initial medium and as a 2% ethanol challenge), and two different types of sugars (glucose or xylose). Table 4 summarizes the most important features of each assay in this data set. The expression levels for 4,290 genes were surveyed for each assay in this study. It is noteworthy to mention that their analysis focused on comparing the two genetic backgrounds, thus trying to establish strain-specific signatures.

**Table 4:** Experimental conditions used to obtain the data set analyzed in "the ethanol response in *E. coli*" case study (see reference [16] for more details).

Assay characteristics				
Assay	Strain	Sugar	1% EtOH in the initial medium	2% EtOH challenge

1	KO11	Glucose	NO	NO
2	LY01	Glucose	NO	NO
3	KO11	Xylose	NO	NO
4	LY01	Xylose	NO	NO
5	KO11	Glucose	YES	NO
6	LY01	Glucose	YES	NO
7	KO11	Xylose	YES	NO
8	LY01	Xylose	YES	NO
9	KO11	Glucose	NO	YES
10	LY01	Glucose	NO	YES
11	KO11	Xylose	NO	YES
12	LY01	Xylose	NO	YES

Gene expression was surveyed for 4,290 genes in a total of 12 different experimental conditions (i.e., assays).

**Figure 10:**
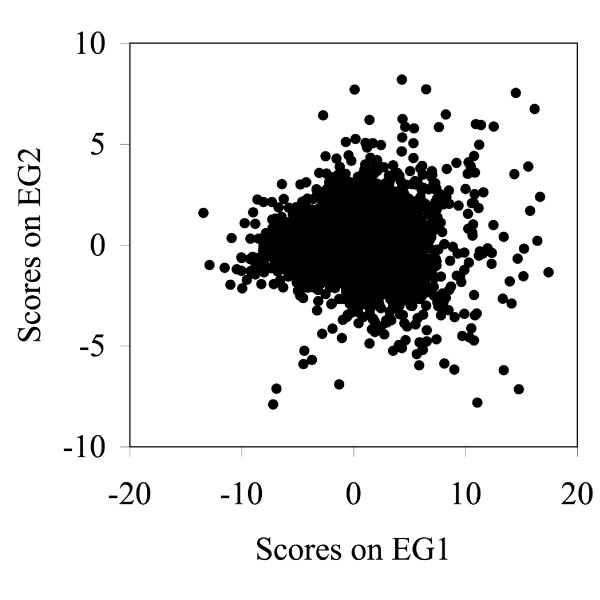
A scores plot of EG2 vs. EG1 for the ethanol data. The genes do not appear to separate into clusters or have structure to facilitate further analysis.

**Figure 11:**
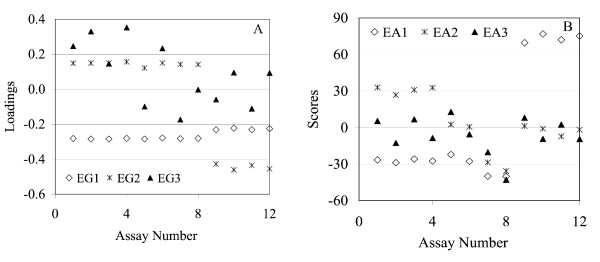
The loading and score plots for EG (A) and EA (B) versus assay number based on the first three PCs for the ethanol data set. The EAs efficiently identify signatures corresponding to ethanol- and non-ethanol-challenged cultures, presence and absence of ethanol in the initial culture medium, and a strain-specific signature and appear to provide better assays grouping than the EGs for the first three PCs.

The two-dimensional score plot with EG2 vs. EG1 is shown in Figure 10. From a visual examination of this plot, there does not appear to be any grouping of genes. Therefore, this appears to be another case where this plot is not useful due to a lack of gene clusters. We apply the PM beginning with Step 1. The EG loading and EA score plots are given in Figure 11 for the first three PCs. EG1, EG2, and EG3 explained about 80%, 13%, and 2% of the total variation, respectively, and EA1, EA2, and EA3 explained about 55%, 13%, and 10%, respectively. Comparison of the two plots suggests that the EA does the best job of grouping the assays. Both EG1 and EG2 separate assays 1–8 from assays 9–12: i.e., distinguish the 2% ethanol-challenged cells (9–12) from the non-ethanol challenged cells (1–8) (see also Table 4). Although EG1 explains about 80% of the total variation it just marginally separated these two groups of cultures. EG3, in a very undistinguishable manner, appears to account for a combination of the effects of strains and type of sugar.

The EA plot (Figure 11B) however distinguishes three different characteristics of the assays in a very clear way. EA1, which explains the largest fraction of the total variation, made a clear distinction between ethanol and non-ethanol-challenged cultures. EA2 on the other hand, further separates non-ethanol challenged cultures into those with no ethanol present in the initial medium (1–4) from those containing 1% ethanol in the initial medium (5–8) (see also Table 4). The latter group is further separated into two cultures containing the sugar glucose (5–6) and two others containing xylose (7–8). Finally, EA3 represents a strain-specific signature as it clearly separates strain KO11 (assays 1, 3, 5, 7, 9, 11) from strain LY01 (2, 4, 6, 8, 10, 12). In summary, the EA-based analysis is clearly superior here, and we therefore chose to do only an EA analysis. In addition, we decided to focus on the results for the first PC (i.e., EA1), which represents the assay signature corresponding to the response of the cultures to a 2% ethanol challenge. Therefore, the assay-specific signature we are exploring is clearly different from the one explored by the study of Gonzalez *et al*. [16]; i.e., while we are studying the ethanol signature their study focused on the strain signature. From the EA score plot in Figure 11B, the non-ethanol scores are all negative and closer together. Similarly, the ethanol scores are all positive and closer together. Therefore, only one PC (EA1) and two assay profiles from this PC are sufficient for determining the gene signatures for ethanol and non-ethanol challenged cultures. The negative scores for EA1 make up the non-ethanol profile and the positive scores make up the ethanol profile.

**Figure 12:**
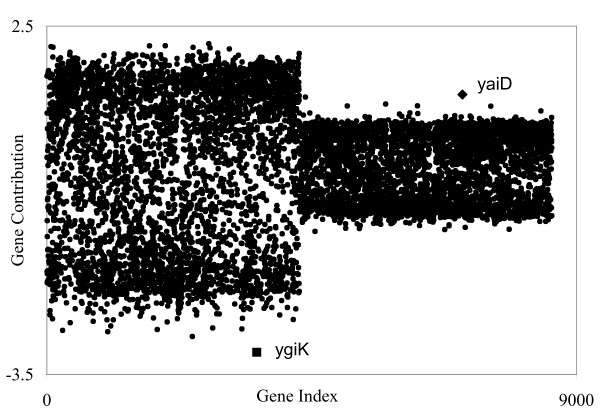
The EA1 contribution plot for the non-ethanol assay group (index numbers 0–4,291) and the ethanol assay group (index numbers 4,292–8,582). The non-ethanol group is more spread out and skewed negatively. The values for the ethanol group are skewed positively. For each group, the top ranking genes are highlighted.

**Figure 13:**
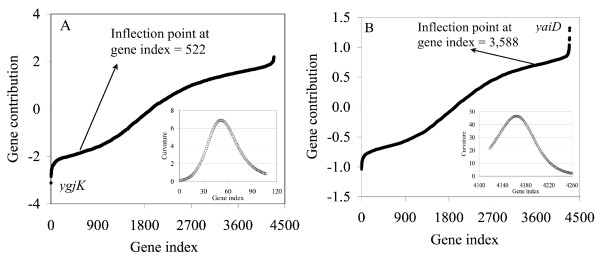
Ranked gene contribution plots for the non-ethanol (A) and ethanol (B) signatures. Complete gene signatures and subset of genes contributing the most to each signature are identified by the existence of points of inflection and maximum curvature, respectively.

In Figure 12 we plot the gene contributions of the non-ethanol assay followed by the ethanol assay against the order of the genes. This plot shows that the non-ethanol profile has about twice as much spread as the ethanol profile. In addition, the non-ethanol spread is skewed towards the negative numbers and the ethanol spread is skewed towards the positive numbers, which explains the negative scores for non-ethanol assays and the positive scores for ethanol assays. The negative values that separate from the non-ethanol cluster make up the gene signature for the non-ethanol. Similarly, the positive values that separate from the ethanol cluster correspond to the gene signature for ethanol. The ranked contribution plots in Figure 13 allow us to find the gene signatures for these two groups of assays under EA1.

As for the two previous case studies, inflection points and sharp regions in the ranked contributions plots in Figure 13 clearly define signature groups. Therefore, the identification of the group of genes composing the ethanol and non-ethanol signatures is straightforward as described in previous sections: i.e., cut-offs are identified by calculating points of inflection and maximum curvature. Using this approach, 702 and 522 genes were identified as part of the complete ethanol and non-ethanol signatures, respectively. In addition, two subsets of genes were identified that contributed the most to each signature (see identification of the point of maximum curvature in insets of Figure 13), which include 126 and 51 genes in the ethanol and non-ethanol signatures, respectively. The top ranked genes are labeled in Figure 13, and Table 5 shows the top ten genes in each signature along with their rank and expression ratios. Note that genes defining the ethanol signature are in fact up-regulated (expression ratios > 1) in response to the ethanol challenge while those composing the non-ethanol signature are down-regulated (expression ratios < -1). Although highly ranked genes frequently exhibit a larger expression ratio, this is not always true because in the PM a gene's contribution is based on product of its loading and expression level and not just its loading. Tables 6 and 7 give the signatures in terms of the genes contributing the most as identified by the points of maximum curvature in Figure 13 [for complete tables, including gene function, see Additional file 2]. We examined the functions encoded by this group of genes (Table 8) and found them very revealing of the metabolic rearrangements associated with the response of the cells to an ethanol challenge.

**Table 5:** Top ten genes in the ethanol (first three columns) and non-ethanol (last three columns) signatures along with their rank and expression ratios.

Gene Name	Rank	Expression Ratio	Gene Name	Rank	Expression Ratio
*yaiD*	1	298.3	*ygjK*	1	-20.9
*argH*	2	170.0	*tktA*	2	-5.2
*mngA*	3	85.6	*dsbC*	3	-8.5
*plsC*	4	76.9	*cvrA*	4	-2.3
*caiA*	5	300.3	*nrfE*	5	-11.9
*yebU*	6	51.6	*yehI*	6	-19.2
*ylbF*	7	29.9	*ybbA*	7	-4.1
*nrfG*	8	46.8	*evgS*	8	-16.2
*yaiY*	9	27.6	*ynfE*	9	-14.4
*pnp*	10	4.9	*pqiB*	10	-3.7

Expression ratios are defined as follows. For the ethanol signature, as the average expression level of a gene in the 4 ethanol-challenged cultures divided by the average expression level in the 8 cultures not exposed to ethanol. For the non-ethanol signature, the ratio was inverted and a negative sign was included to indicate that these genes are down-regulated as the result of the ethanol challenge.

**Table 6:** The 126 genes contributing the most to the ethanol signature as defined by the maximum curvature in Figure 13 A.

Gene Name	Rank	Expression Ratio	Gene Name	Rank	Expression Ratio	Gene Name	Rank	Expression Ratio
*yaiD*	1	298.3	*uidR*	43	31.2	*yagR*	85	5.7
*argH*	2	170.0	*hcaR*	44	60.6	*mtlA*	86	2.3
*mngA*	3	85.6	*yciQ*	45	1.4	*ydhS*	87	2.7
*plsC*	4	76.9	*nagC*	46	4.1	*ybgH*	88	3.7
*caiA*	5	300.3	*yqcD*	47	4.3	*ygfZ*	89	3.5
*yebU*	6	51.6	*ynfA*	48	1.8	*aat*	90	8.7
*ylbF*	7	29.9	*glvC*	49	3.2	*speD*	91	1.7
*nrfG*	8	46.8	*yiaL*	50	14.0	*yagZ*	92	6.2
*yaiY*	9	27.6	*dbpA*	51	2.3	*yaeQ*	93	1.6
*pnp*	10	4.9	*ydhB*	52	14.3	*uhpT*	94	9.4
*mepA*	11	10.5	*grxB*	53	2.1	*yidF*	95	12.1
*amtB*	12	3.7	*hnr*	54	4.9	*ydiF*	96	2.0
*xthA*	13	22.6	*cbl*	55	46.8	*ydiU*	97	2.0
*ybfL*	14	5.0	*hemX*	56	2.2	*yhaM*	98	22.2
*moaE*	15	2.7	*fliG*	57	30.3	*ycgE*	99	8.4
*fdrA*	16	10.0	*yhaJ*	58	14.3	*ycdJ*	100	3.4
*topB*	17	2.9	*ydaP*	59	9.3	*ydfE*	101	3.6
*nlpC*	18	9.6	*tolB*	60	2.1	*yfcD*	102	7.3
*yagX*	19	2.1	*yagB*	61	4.8	*ytfM*	103	2.0
*ydcK*	20	21.8	*livJ*	62	1.9	*ylbC*	104	3.9
*yhfR*	21	18.3	*ybgI*	63	2.4	*rstA*	105	7.3
*fliF*	22	58.3	*tus*	64	10.5	*ycdI*	106	6.5
*zipA*	23	4.4	*uvrY*	65	4.9	*sfcA*	107	7.5
*ybhD*	24	29.9	*yhhX*	66	8.2	*ynjH*	108	1.9
*fhuA*	25	3.8	*sgbU*	67	5.7	*ybjN*	109	11.8
*ycfX*	26	52.4	*ydfP*	68	3.2	*ynfC*	110	2.9
*xerC*	27	5.7	*lysR*	69	7.6	*yidP*	111	29.8
*yheH*	28	4.4	*yneB*	70	6.5	*ybfO*	112	2.1
*wzzB*	29	4.1	*tktB*	71	3.2	*ydcI*	113	34.6
*yeeY*	30	25.2	*yhiP*	72	3.1	*ybdL*	114	3.0
*yiaJ*	31	18.3	*yidL*	73	6.8	*yggF*	115	13.5
*uvrC*	32	2.2	*yfeA*	74	8.2	*gcvA*	116	3.0
*ychA*	33	16.9	*yjcZ*	75	3.3	*yibN*	117	3.6
*hyfD*	34	2.7	*ycbX*	76	5.3	*cpsB*	118	2.4
*kdgR*	35	3.6	*yhcP*	77	2.2	*nagA*	119	3.4
*ydfU*	36	2.57	*recX*	78	36.5	*mhpB*	120	19.8
*yfeR*	37	46.5	*nanR*	79	5.5	*livK*	121	6.9
*parC*	38	2.5	*yccU*	80	2.1	*mnmE*	122	1.7
*yhiI*	39	12.2	*hyuA*	81	6.2	*fes*	123	3.9
*ygbI*	40	38.1	*nadB*	82	1.7	*pspF*	124	3.4
*ypdC*	41	29.9	*yaeJ*	83	2.4	*ygcP*	125	83.3
*yeiQ*	42	13.5	*ybhS*	84	3.9	*intA*	126	2.5

Expression ratios are defined as the average expression level of a gene in the 4 ethanol-challenged cultures divided by the average expression level in the 8 cultures not exposed to ethanol.

**Table 7:** The 51 genes contributing the most to the non-ethanol signature as defined by the maximum curvature in Figure 13 B.

Gene Name	Rank	Expression Ratio	Gene Name	Rank	Expression Ratio	Gene Name	Rank	Expression Ratio
*ygjK*	1	-20.9	*nagE*	18	-4.8	*ygfK*	35	-6.6
*tktA*	2	-5.2	*kdpB*	19	-4.0	*trpD*	36	-8.7
*dsbC*	3	-8.5	*tufA*	20	-2.0	*gshA*	37	-1.8
*cvrA*	4	-2.3	*alsE*	21	-14.8	*yejA*	38	-4.7
*nrfE*	5	-11.9	*aidB*	22	-4.3	*rplX*	39	-2.8
*yehI*	6	-19.2	*fusA*	23	-2.9	*ygbE*	40	-7.9
*ybbA*	7	-4.1	*ybhH*	24	-5.7	*yqjI*	41	-4.2
*evgS*	8	-16.2	*ybeQ*	25	-6.3	*glnS*	42	-2.1
*ynfE*	9	-14.4	*rplO*	26	-5.0	*yoaD*	43	-240.1
*pqiB*	10	-3.7	*betT*	27	-2.4	*cyaA*	44	-4.4
*bglA*	11	-2.0	*leuA*	28	-4.1	*amiA*	45	-3.4
*dnaN*	12	-1.7	*norV*	29	-2.9	*lon*	46	-6.4
*feoB*	13	-25.9	*deaD*	30	-2.2	*yhgF*	47	-3.4
*yphG*	14	-7.0	*tufB*	31	-1.8	*hscC*	48	-22.1
*arcB*	15	-10.4	*gppA*	32	-4.8	*infC*	49	-1.8
*rne*	16	-6.9	*alaS*	33	-5.0	*yfiE*	50	-5.8
*yehU*	17	-3.8	*rplD*	34	-7.7	*rplP*	51	-4.7

Expression ratios are calculated by dividing as the average expression level of a gene in the 8 cultures not exposed to ethanol by the average expression level in the 4 ethanol-challenged cultures. A negative sign has been included to indicate that these genes are down-regulated as the result of the ethanol challenge.

**Table 8:** The genes composing the complete ethanol and non-ethanol signatures (see Tables 6 and 7) are organized here by functional groups as described by Riley and Serres [20].

**Functional Group**	**Gene Name**
Known Functions (92, 100%)	

Carbon compound catabolism (4/4%)	*nagA mhpB; bglA alsE*
Central intermediary metabolism (6/7%)	*tktB speD ylbC sfcA cpsB; tktA*
Energy metabolism (3/3%)	*nrfG hyfD; nrfE*
Amino acid biosynthesis (3/3%)	*argH; leuA trpD*
Cofactors and prosthetic groups (6/7%)	*moaE grxB hemX nadB; gshA*
Lipid metabolism (1/1%)	*plsC*
Transport (13/14%)	*mngA amtB glvC livJ mtlA uhpT livK fes; cvrA feoB nagE kdpB betT*
Cell processes (10/11%)	*fliF zipA xerC fliG tolB; pqiB aidB norV amiA hscC*
Cell structure (5/5%)	*mepA nlpC fhuA ynfA; dsbC*
Regulatory function (15/16%)	*wzzB uidR hcaR nagC hnr cbl lysR rstA gcvA pspF; evgS arcB gppA cyaA lon*
DNA replication, repair, modification (7/8%)	*yaiD xthA topB uvrC parC tus; dnaN*
Transcription/RNA processing/degradation (4/4%)	*pnp dbpA; rne deaD*
Translation/post-translational modification (12/13%)	*aat mnmE; tufA fusA rplO tufB alaS rplD rplX glnS infC rplP*
Phage, transposon, or plasmid (3/3%)	*ydaP intA ylbF*
Putative Functions (59, 100%)	
General (21/36%)	*yfeA ycbX ydhS yibN yagX yeiQ yiaL yhhX yneB yccU ycdJ yhiI yciQ yggF ycdI ybdL ybfL; ygjK ynfE yphG ygbE*
Cell processes an energy metabolism (1/2%)	*caiA*
Cell structure (1/2%)	*yebU*
Central intermediary metabolism (1/2%)	*sgbU*
Carbon catabolism (1/2%)	*ydiF*
Nucleotide biosynthesis (1/2%)	*hyuA*
Transport (7/12%)	*fdrA yheH yhiP ybhS ybgH; ybbA yejA*
Regulation (26/44%)	*ygfZ yhfR ybhD ycfX yeeY yiaJ kdgR yfeR ygbI ypdC ydhB yhaJ uvrY yidL recX nanR yidF ycgE yfcD ydcI ygcP ybjN yidP; yehI yehU yfiE*
Hypothetical Functions (26, 100%)	*yaiY ydcK ychA ydfU yqcD yagB ybgI ydfP yjcZ yhcP yaeJ yagR yagZ yaeQ ydiU yhaM ydfE ytfM ynjH ynfC ybfO; ybhH ybeQ yqjI yoaD yhgF*

A semicolon separates the two groups of genes and those corresponding to the non-ethanol signature are underlined. The composition of the groups was updated using the latest information available in the literature [21-43]. Functional groups were organized in three categories: (1) known functions, (2) putative functions, and (3) hypothetical functions. The putative category was subdivided according to the specific putative function of each gene. Numbers in parenthesis are the total number of genes identified for the functional group and the percentage they represent respect to the total number of genes identified for the category, respectively.

As expected for the immediate response to an environmental challenge, the cells significantly modified functions involved in regulatory, transport, and general processes (i.e., the largest changes in the "known functions" category in Table 8 are observed for these three functional groups), and important changes appear to take place at the translation and post-translational modification levels. The same trend was observed for the "putative functions" categories, where most populated functions were those involved in regulation and transport. The group of genes composing these signatures also provides very important information to formulate hypothesis about which specific gene(s)/function(s) are involved in the cellular response to an ethanol challenge. For example, many of the top-ranked genes (Table 5) encode functions that one would expect to be involved in the cellular response to an ethanol challenge such as the metabolism and transport of osmolytes (*mngA*, *cvrA*, and *caiA*), the biosynthesis of phospholipids (*plsC*) which are major constituents of the cell membrane, and the repairing of misfolded proteins (*dsbC*). In fact, increased tolerance to ethanol in certain *E.coli *strains is related to the increased availability of osmolytes like betaine and trehalose [16,19]. In summary, the application of the PM to this data set allowed the identification of a group of genes representing the ethanol signature, which can be hypothesized to be involved in the cellular response to an ethanol challenge. Such hypothesis form the basis of current studies in our groups aimed at elucidating the mechanisms/processes involved in the response to ethanol in *E. coli*.

## Conclusion

This article proposes a new and powerful PCA-based method for the identification of assay-specific gene signatures of ranked order in the analysis of FG data. This method is unique for several reasons. First, it is the only one, to our knowledge, that uses gene contribution, a product of the loading and expression level, to obtain assay signatures. Our proposed method (PM) develops and exploits two types of assay-specific contribution plots. To our knowledge the development and use of these plots is new to the application of PCA in the FG area. The first type plots the assay-specific gene contribution against the given order of the genes and reveals variations in distribution between assay-specific gene signatures as well as outliers within assay groups indicating the degree of importance of the most dominant genes. The second type plots the contribution of each gene in ascending or descending order against a constantly increasing index. This type of plots reveals assay-specific signatures defined by the inflection points in the curve. In addition, sharp regions within the gene signature in the ranked contribution plots define the genes that contribute the most to the signature. We proposed and used the curvature as an appropriate metric to characterize sharp regions of these plots, thus identifying the subset of genes contributing the most to the signature. Secondly, we know of no other method that selects an assay group by comparing EG loadings against the assays and EA scores against the assays. It is worth noting that no set of PCs (EGs or EAs) will necessarily contain all the information for grouping assays. For example, for the human tissue data in our second study, although EA2 and EA3 were best for obtaining the muscle, brain and liver signatures, the EGs provided better assay groupings for Colon 5; Lungs 1 and 2; Lungs 4 and 5; Stomach 1; Breast 7 and 9; Kidneys 7–10; Placenta 2 and 3; Vulva "*", 1 and 2; Blood; and Cervix 2. As mentioned earlier, these signatures are available at the website:  . Finally, the PM uses the full dataset to determine the final gene signature, thus eliminating the chance of gene exclusion by poor screening in earlier steps.

This article presented the PM in six basic steps and applied it to three different case studies: one artificial and two real data sets. In the artificial data study, which consisted of 1,600 noisy genes, 200 noisy sinusoidal genes, and 200 noisy exponential genes, the PM identified all 400 genes with patterns in a sinusoidal/exponential-specific gene signature and most of the genes with sinusoidal patterns in a sinusoidal-specific gene signature. In the first study involving real data, we compared the results and methodology of the PM with another one (the method of Misra *et al*. [13]) of similar ability (i.e., a PCA-based method) in a study of real DNA microarray data. Their approach represents the most refined used of PCA for the analysis of FG data that we have found in the literature. The tissue-specific signatures identified by the PM not only included the genes previously identified by Misra *et al*. [13] but also added genes that are known to be linked to the specific tissue and other of unknown connection, thus establishing hypothesis regarding their potential involvement in the functions of those tissues. In the second study involving real data, the ethanol case, we were able to explore a data set that had not been previously analyzed using any PCA-based approach. The PM was able to identify different assay-specific signatures including the ethanol- and strain-signature. A detailed study of the ethanol signature using the PM resulted in the clear identification of a group of genes that are relevant to the response of *E. coli *to an ethanol challenge. These findings confirm the capability of the PM to generate testable hypothesis that will contribute to elucidating different biological processes (in this example the basis of the response of the cells to an ethanol challenge).

## Abbreviations

PCA, principal component analysis; FG, functional genomic(s); EG, eigengene; PM, proposed method; EA, eigenassay; PC, principal component.

## Authors' contributions

D.K.R. and R.G. designed research; D.K.R., R.G., D.Z., A.L.J., A. M., and J.W.G. performed research; D.K.R., R.G., J.W.G., and A. M. wrote the paper.

## Supplementary Material

Additional File 1 Human tissue expression data case study. Data sets used in the "human tissue expression data case study". Click here for file

Additional File 2 Gene signatures for "case studies". Complete gene signatures, including gene names and a brief description of their function, are provided for the "human tissue expression data case study" and "the ethanol response in *E. coli *case study". Click here for file

Additional File 3 Ethanol response in *E. coli *case study. Data sets used in the "ethanol response in *E. coli *case study". Click here for file
